# Patterns of gray matter atrophy in genetic frontotemporal dementia: results from the GENFI study

**DOI:** 10.1016/j.neurobiolaging.2017.10.008

**Published:** 2018-02

**Authors:** David M. Cash, Martina Bocchetta, David L. Thomas, Katrina M. Dick, John C. van Swieten, Barbara Borroni, Daniela Galimberti, Mario Masellis, Maria Carmela Tartaglia, James B. Rowe, Caroline Graff, Fabrizio Tagliavini, Giovanni B. Frisoni, Robert Laforce, Elizabeth Finger, Alexandre de Mendonça, Sandro Sorbi, Martin N. Rossor, Sebastien Ourselin, Jonathan D. Rohrer, Genetic FTD Initiative, GENFI

**Affiliations:** a Dementia Research Centre, Department of Neurodegenerative Disease, UCL Institute of Neurology, London, UK; b Centre for Medical Image Computing, University College London, London, UK; c Erasmus Medical Center, Rotterdam, the Netherlands; d Centre for Ageing Brain and Neurodegenerative Disorders, Neurology Unit, University of Brescia, Brescia, Italy; e Department of Pathophysiology and Transplantation, “Dino Ferrari” Center, University of Milan, Fondazione Cà Granda, IRCCS Ospedale Maggiore Policlinico, Milan, Italy; f Cognitive Neurology Research Unit, Sunnybrook Health Sciences Centre, Hurvitz Brain Sciences Research Program, Sunnybrook Research Institute, Department of Medicine, University of Toronto, Toronto, Ontario, Canada; g Tanz Centre for Research in Neurodegenerative Diseases, University of Toronto, Toronto, Ontario, Canada; h Department of Clinical Neurosciences, University of Cambridge, Cambridge, UK; i Karolinska Institutet, Department NVS, Center for Alzheimer Research, Division of Neurogeriatrics, Huddinge, Sweden; j Department of Geriatric Medicine, Karolinska University Hospital, Stockholm, Sweden; k Istituto Neurologico Carlo Besta, Milan, Italy; l IRCCS San Giovanni di Dio Fatebenefratelli, Brescia, Italy; m Université Laval, Quebec, Canada; n University of Western Ontario, Ontario, Canada; o Faculdade de Medicina, Universidade de Lisboa, Lisbon, Portugal; p Department of Neurosciences, Psychology, Drug Research and Child Health (NEUROFARBA), University of Florence, Florence, Italy; q IRCCS Don Gnocchi, Firenze, Italy

**Keywords:** Frontotemporal dementia, Magnetic resonance imaging, Atrophy, Voxel-based morphometry, Preclinical dementia

## Abstract

Frontotemporal dementia (FTD) is a highly heritable condition with multiple genetic causes. In this study, similarities and differences of gray matter (GM) atrophy patterns were assessed among 3 common forms of genetic FTD (mutations in *C9orf72*, *GRN*, and *MAPT*). Participants from the Genetic FTD Initiative (GENFI) cohort with a suitable volumetric T1 magnetic resonance imaging scan were included (319): 144 nonmutation carriers, 128 presymptomatic mutation carriers, and 47 clinically affected mutation carriers. Cross-sectional differences in GM volume between noncarriers and carriers were analyzed using voxel-based morphometry. In the affected carriers, each genetic mutation group exhibited unique areas of atrophy but also a shared network involving the insula, orbitofrontal lobe, and anterior cingulate. Presymptomatic GM atrophy was observed particularly in the thalamus and cerebellum in the *C9orf72* group, the anterior and medial temporal lobes in *MAPT*, and the posterior frontal and parietal lobes as well as striatum in *GRN*. Across all presymptomatic carriers, there were significant decreases in the anterior insula. These results suggest that although there are important differences in atrophy patterns for each group (which can be seen presymptomatically), there are also similarities (a fronto-insula-anterior cingulate network) that help explain the clinical commonalities of the disease.

## 1 Introduction

Frontotemporal dementia (FTD) is a common cause of dementia with around one-third of cases being familial, most commonly caused by mutations in 1 of 3 genes: chromosome 9 open reading frame 72 (*C9orf72*), progranulin (*GRN*), and microtubule-associated protein tau (*MAPT*) (Rohrer et al., 2009). Studying mutation carriers in the years before any signs of clinical manifestation provides insight into the early stage of the disease process. One biomarker of particular interest in presymptomatic FTD is brain atrophy as measured by structural magnetic resonance imaging (MRI). Although there have been many studies describing the atrophy patterns in FTD (Mahoney et al., 2012, Rohrer et al., 2010, Whitwell et al., 2009, Whitwell et al., 2012), these have commonly been smaller single-site studies of patients who are already symptomatic. The multicenter Genetic FTD Initiative (GENFI) investigates both affected and at-risk FTD family members, with preliminary region of interest (ROI) based cross-sectional analysis indicating that carriers have significantly lower cortical and subcortical volumes a number of years before the expected age at onset (Rohrer et al., 2015). In this study, we use data from GENFI to perform a whole-brain voxel-wise analysis to provide complementary information to the previous ROI study and expand on previous voxel-based morphometry studies in genetic FTD, with a particular focus on comparing and contrasting patterns of atrophy between the mutations and determining the extent of gray matter loss in the presymptomatic phase.

## 2 Material and methods

### 2.1 Participants

At the time of the second data freeze in the GENFI study, 365 participants had been recruited across 13 centers in the United Kingdom, Canada, Italy, Netherlands, Sweden, and Portugal, of whom 319 had a usable volumetric T1-weighted MRI scan for analysis (15 participants did not have a scan, and a further 31 participants were excluded as the scans were of unsuitable quality due to motion, other imaging artifacts, or pathology unlikely to be attributed to FTD). All participants were known to be a symptomatic carrier of a pathogenic mutation in *C9orf72*, *GRN*, or *MAPT* or to be an at-risk first-degree relative. Patients were considered symptomatic when the assessing clinician felt that the patient had evidence of progressive cognitive or behavioral change. All participants underwent genetic testing to determine whether they were a carrier or noncarrier: in total, 144 were noncarriers, 128 were presymptomatic mutation carriers, and 47 were affected mutation carriers (Table 1). All participants underwent a standardized clinical assessment as described previously (Rohrer et al., 2015). All aspects of the study were approved by the institutional review boards for each of the GENFI sites, with every participant providing written informed consent.

**Table 1:** Demographics of participants included in the analysis

Variable	Noncarriers	C9orf72		GRN		MAPT		*p*-value		
		Presymptomatic	Affected	Presymptomatic	Affected	Presymptomatic	Affected	All groups	At-risk groups (including noncarriers)	Affected groups
N	144	40	25	65	12	23	10	-	-	-
Age, mean (SD)	48.7 (14.3)	43.5 (10.5)	65.2 (7.7)	48.9 (10.7)	63.2 (6.0)	38.6 (9.0)	57.2 (5.9)	**<0.001**	**<0.001**	**0.011**
%Female	63	63	28	63	67	61	30	**0.021**	1.000	0.083
EYO, mean (SD)	−10.5 (14.2)	−15.0 (12.5)	5.8 (5.0)	−10.3 (11.3)	1.4 (2.1)	−11.8 (10.3)	5.9 (3.5)	**<0.001**	0.270	**0.001**
Education, y	13.8 (3.4)	13.9 (3.0)	13.1 (4.5)	14.1 (3.1)	10 (3.8)	13.4 (3.4)	12.2 (4.7)	**0.040**	0.788	0.125
Disease duration, y	N/A	N/A	6.6 (4.8)	N/A	2.5 (1.3)	N/A	4.9 (5.3)	N/A	N/A	**0.010**
MMSE (max = 30)	29.2 (1.3)	29.2 (1.2)	24.8 (4.2)	29.1 (1.4)	20.7 (6.2)	29.4 (1.4)	24.7 (4.9)	**<0.001**	0.359	0.139

Diagnoses in affected subjects: bvFTD 33 (18 *C9orf72*, 5 *GRN*, 10 *MAPT*), 3 FTD-ALS (all *C9orf72*), 7 nonfluent variant primary progressive aphasia (PPA) (2 *C9orf72*, 5, *GRN*), 1 semantic variant PPA (*C9orf72)*, 1 corticobasal syndrome (*GRN*), 1 dementia - not otherwise specified (*C9orf72*).

Bold text indicates a statistically significant difference (*p*< 0.05) between groups.

### 2.2 MR image acquisition

Participants underwent a 1.1-mm isotropic resolution volumetric T1 MR imaging on a 3T scanner (10 sites: 5 Siemens Trio, 1 Siemens Skyra, 3 Philips Achieva, 1 GE Discovery MR750) or 1.5T scanner (1–1.25 mm isotropic resolution) when a 3T scanner was not available (3 sites: Siemens Avanto, Siemens Aera, GE Signa HDxt).

All analyses were performed using the Statistical Parametric Mapping toolbox (SPM12) in Matlab. The images were first segmented into maps representing probability of gray matter, white matter, and cerebrospinal fluid at each voxel (Ashburner and Friston, 2005). Next, all images were spatially normalized using geodesic shooting (Ashburner and Friston, 2011) to a study specific template, modulating the probability maps to preserve tissue volumes. The warped and modulated tissue maps were smoothed with a Gaussian kernel of full width at half max of 6 mm to reduce errors caused by misalignment while at the same time allowing for detection of differences over small regions of the brain. Analysis was limited to a gray matter mask that included voxels where the mean probability of the gray matter mask over all subjects was 0.2 or above. Estimates of total intracranial volume were computed by summing the 3 tissue class volumes (Malone et al., 2014).

### 2.3 Statistical analysis

Baseline demographic variables were compared across groups using a Kruskal-Wallis test for the continuous variables and a Fisher's exact test for gender. Three tests were performed: across all groups, across the at-risk groups (noncarriers and presymptomatic mutation carriers), and across the affected groups.

Data at each voxel of the smoothed, warped, and modulated gray matter (GM) maps were fitted to a general linear model. We implemented 2 models, subdividing the carriers into more distinct subgroups with each level. In model 1, a 3-level factor was used: nonmutation carriers, presymptomatic mutation carriers, and affected carriers. Model 2 further subdivided the carrier subgroup to create a 7-level factor: noncarriers, presymptomatic and affected *C9orf72* carriers, presymptomatic and affected *GRN* carriers, and presymptomatic and affected *MAPT* carriers. All models included a factor variable for imaging site and covariates for age, gender, and total intracranial volume. As it was expected that members from the same family enrolled in GENFI might have covariance in brain structure, family membership was included in the model as a random effect.

Contrasts were performed to look at the following pairwise differences: in model 1, noncarriers with presymptomatic carriers and noncarriers with affected carriers; and in model 2, noncarriers with each of the 6 carrier groups, as well as comparisons between the 3 mutations within the presymptomatic and affected groups separately. In model 2, we combined the 3 contrasts between noncarriers and each of the affected carrier subgroups to construct a compound hypothesis, where the null hypothesis was that 2 or less contrasts were significant. Significant findings from the compound hypothesis indicate GM atrophy patterns that are common to all 3 genetic mutations. To correct for the multiple comparisons problem inherent in mass univariate statistical analysis, we controlled for voxel-level family-wise error (FWE) at *p* < 0.05. If results did not reach significance after correction for multiple comparisons, we describe patterns at an uncorrected level of *p* < 0.001. Findings were reported with a cluster extent greater than the empirically determined threshold provided by SPM (the expected voxels per cluster, k = 56 voxels, 190 mm^3^).

## 3 Results

### 3.1 Demographics variables

Baseline demographic variables are shown in Table 1. Across all groups, there are significant differences in all variables. These findings are primarily driven by differences between the at-risk groups and the affected groups. When comparing the at-risk groups, only significant differences in age remained, with the noncarriers and presymptomatic *GRN* carriers being older than the *C9orf72* and *MAPT* carriers. When comparing only the affected groups, differences remained in age, estimated years to onset, and the disease duration as determined by the participant's actual onset. *MAPT* carriers were younger than the other 2 mutations, while affected *GRN* carriers had lower estimated years to onset and disease duration.

### 3.2 Symptomatic mutation carriers

Comparison of all affected mutation carriers (*GRN*, *MAPT,* and *C9orf72* combined) with noncarriers shows widespread decrease in GM, with the most significant areas in the orbitofrontal and inferior frontal lobes, temporal lobes (anterior > posterior), insula, anterior cingulate, parietal lobe (around the precuneus), and cerebellum (eTable 1 and eFig. 1).

**Fig. 1:**
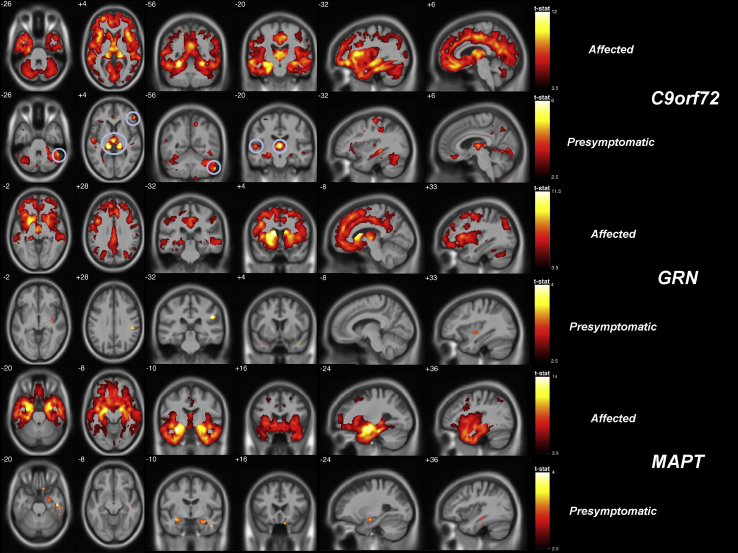
Gray-matter (GM) differences by mutation and clinical status. GM differences in affected (odd rows, *p* < 0.05 FWE-corrected) and presymptomatic (even rows, *p* < 0.001 uncorrected) carriers compared to noncarriers. Comparisons to the *C9orf72* carriers are in the top 2 rows (with findings at *p* < 0.05, FWE-corrected circled in the presymptomatic group), the *GRN* carriers in the middle 2 rows, and *MAPT* carriers in the bottom 2 rows.

**Fig. 2:**
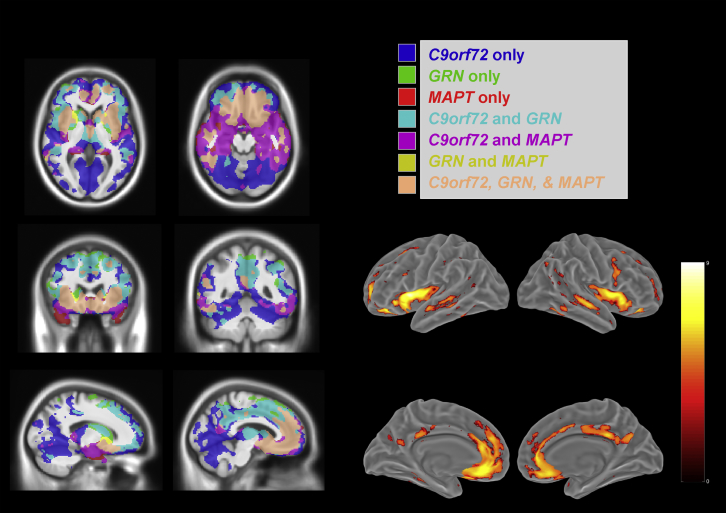
Comparison of gray matter atrophy patterns across the 3 genetic mutations. Comparison of atrophy patterns across the 3 genetic groups (symptomatic carriers). On the left hand of the figure, masks of the regions where there are significant differences (*p* < 0.05, FWE-corrected) are shown, color coded by mutation, along with areas where the patterns intersect within 2 or more mutations. The region satisfying the compound hypothesis of all 3 contrasts being true, indicating the intersection of atrophy in these mutations, is coded in light pink. A surface rendering of this intersection is shown on the right. (For interpretation of the references to color in this figure legend, the reader is referred to the Web version of this article.)

Looking at the affected mutation carriers in each individual mutation, different but overlapping patterns of atrophy were seen (eTable 2, Fig. 1, Fig. 2). In *C9orf72* carriers, there were significant areas of GM loss throughout the brain including the frontal (orbitofrontal > dorsolateral/ventromedial prefrontal), temporal (inferior > superior), insula (anterior > posterior), and cingulate (both anterior and posterior) regions as well as more posterior cortical areas (precuneus and inferior > superior parietal regions). Subcortical structures were particularly affected, most significantly the thalamus but also the hippocampus, amygdala, and basal ganglia. GM loss was also seen in the cerebellum, affecting the superior posterior area most significantly (Fig. 1). Significant GM atrophy was seen in the affected *GRN* carriers in the frontal lobe (particularly in the dorsolateral and ventromedial prefrontal cortices), insula, anterior cingulate, superior and middle temporal gyri and striatum (caudate and putamen) as well as more posteriorly in the lateral and medial parietal lobes (precuneus). Affected *MAPT* carriers had significant atrophy in the hippocampus, amygdala, and temporal lobes (particularly anterior, medial, and inferior) as well as the insula and orbitofrontal lobe. Direct comparisons between affected mutation groups are shown in eTable 2.

Fig. 2 summarizes the similarities and differences in GM atrophy within the 3 mutations. The blue (*C9orf72*), green (*GRN*), and red (*MAPT*) regions are areas where only that specific mutation had evidence of significant GM atrophy in the affected carriers (*p* < 0.05, FWE-corrected). Thalamic, superior cerebellar and very posterior cortical involvement (particularly inferior parietal lobe) is unique to *C9orf72*; superior aspect of the dorsolateral frontal cortex, dorsolateral caudate, and superior parts of the medial parietal regions are only affected in *GRN*; and anteroinferior temporal lobe involvement is unique to *MAPT*. In contrast, the findings from the compound hypothesis indicate the atrophy pattern common to all 3 mutations (light pink), which comprises a network of regions encompassing mainly the insula, orbitofrontal lobe, and anterior cingulate bilaterally.

### 3.3 Presymptomatic mutation carriers

When comparing the combined group of presymptomatic carriers to noncarriers, significant areas of GM loss are seen in the anterior insula when correcting for multiple comparisons (eTable 1), with further regions (uncorrected for multiple comparisons) of GM loss in the orbitofrontal lobe, anterior temporal lobe, posterior insula, parietal lobe and thalamus.

In the individual subgroups of presymptomatic mutation carriers (in comparison with noncarriers) the areas of GM atrophy were similar to those seen in the affected cases but to a lesser extent (Fig. 1, eTable 3). In *C9orf72* carriers there were significant areas of GM loss bilaterally in the thalamus, right superior posterior cerebellum (Crus I), superior temporal and inferior frontal regions when correcting for multiple comparisons, with more extensive loss in the same areas as well as the anterior insula, temporal (including hippocampi and amygdala), and parietal (particularly inferiorly) regions at an uncorrected *p-*value of <0.001 (as shown in Fig. 1). In *GRN* and *MAPT* carriers no areas survived correction for multiple comparisons, but at an uncorrected significance level, areas of GM atrophy were seen in *GRN* carriers in the insula, parietal, posterior frontal and anterior temporal lobes as well as the striatum (Fig. 1, eTable 3), and in *MAPT* carriers in the anterior and medial temporal lobes (including hippocampus and amygdala) and the orbitofrontal lobe (Fig. 1, eTable 3). Direct comparisons between mutation groups are shown in eTable 3.

As there have been reports in the familial AD literature of increased volume in brain structures prior to atrophy (Fortea et al., 2010, Lee et al., 2012), we performed reverse contrasts looking for areas where any of the carrier groups had more GM than the noncarriers. In each of these tests, there were no findings of GM increase, even at an uncorrected level of *p* < 0.001, within any affected or presymptomatic group.

## 4 Discussion

Using a whole-brain voxel-wise analysis of gray matter volume in a genetic FTD cohort, we show evidence of unique areas of atrophy in each mutation with an intersecting region of atrophy affecting all 3 mutation groups in the insula, orbitofrontal lobe, and anterior cingulate. Analysis of presymptomatic carriers shows evidence of atrophy before symptom onset in each of the mutation groups. This work expands on previous studies, which have investigated smaller cohorts, commonly at a single site and primarily focusing on differences in affected patients.

Findings in symptomatic mutation carriers are consistent with atrophy signatures found in previous voxel-based morphometry studies of genetic FTD (Mahoney et al., 2012, Rohrer et al., 2010, Whitwell et al., 2009, Whitwell et al., 2012). In *MAPT* mutation carriers, atrophy primarily affects the anterior and medial temporal lobes, orbitofrontal lobe and insula; in *GRN* mutation carriers atrophy is found in the dorsolateral and ventromedial prefrontal, superolateral temporal and lateral parietal lobes as well as the anterior cingulate, insula, precuneus and striatum; whilst in *C9orf72* mutation carriers there is relatively widespread cortical atrophy including posterior areas, and particularly affecting the thalamus and superoposterior cerebellum. Importantly, in this study, we show through a compound hypothesis of contrasts in the general linear model that there are common regions of atrophy across all 3 genes in the orbitofrontal lobe, insula, and anterior cingulate. Interestingly, this area overlaps substantially with the so-called salience network (Seeley et al., 2007), an intrinsic connectivity network, described as being fundamentally involved in FTD (Filippi et al., 2013, Seeley et al., 2009, Zhou et al., 2010). It is this neuroanatomical correspondence, which likely accounts for the overlapping behavioral clinical syndrome seen in the majority of cases of genetic FTD.

The findings in the presymptomatic carriers add to the region of interest results in the initial GENFI study and other presymptomatic studies (Bocchetta et al., 2016, Rohrer et al., 2015, Whitwell et al., 2012). The strongest evidence of presymptomatic atrophy was observed in *C9orf72* carriers in the thalamus and superoposterior cerebellum. A recent study of 15 presymptomatic *C9orf72* carriers (Lee et al., 2017) also found decreased GM compared to noncarriers in the left thalamus but did not show any differences in the cerebellum. Uncorrected findings of atrophy were observed in *MAPT* mutation carriers in the anteromedial temporal and orbitofrontal lobes, and in striatum, frontal, temporal, and parietal areas of *GRN* carriers. However, when pooling all presymptomatic carriers in model 1, there was significant evidence of atrophy surviving FWE correction for multiple comparisons in the right anterior insula. These results indicate that there may be some distinct regions in which the disease process starts, but those common sites across all 3 mutations may also be involved during the presymptomatic phase of the disease.

There is relatively minimal atrophy in presymptomatic *GRN* carriers. The explanation for this finding is unclear. It is unlikely to represent a difference in the age or expected proximity to onset of the *GRN* presymptomatic cohort compared to the other genes but could be indicative of atrophy occurring nearer to symptom onset in *GRN-*related FTD. A longitudinal study of cortical thickness in 16 presymptomatic *GRN* carriers (Caroppo et al., 2015) found no cross-sectional differences at baseline compared to 17 noncarriers, but longitudinal changes over 20-month follow-up were observed in 1 cluster in the middle and inferior temporal gyrus. This cluster was not found in our presymptomatic *GRN* carriers, but it was present in the affected participants. This could be consistent with the pathophysiological process that occurs in *GRN*, where an additional insult or injury (superimposed on low progranulin levels) is likely to be required to start the neurodegeneration process with more rapid GM loss (and sooner onset of symptoms) subsequently (Martens et al., 2012, Rohrer et al., 2010, Yin et al., 2009). It could also be in part due to the noted asymmetric atrophy in these cases, which can variably affect the right or left hemisphere (Le Ber et al., 2008, Rohrer et al., 2010). In *MAPT* mutation carriers, the findings in the presymptomatic group closely mirror those found in the affected carriers (Fig. 2, bottom 2 rows), and although not reaching significance once correcting for multiple comparisons, the pattern is similar to that of symptomatic cases but to a lesser extent.

In all 3 at-risk groups, it will be important to investigate the GENFI cohort in more detail when a larger cohort is available who can be further stratified by age (or expected time to symptom onset). The current presymptomatic cohort represents a heterogeneous sample with 39 (9 *C9orf72*, 22 *GRN*, 8 *MAPT*) of 130 participants within 5 years of their expected age at onset, when atrophy is more likely to be present, while 33 participants (12 *C9orf72*, 15 *GRN*, and 6 *MAPT*) are more than 20 years away from expected onset, where atrophy should be minimal.

In summary, we were able to observe distinct but overlapping patterns of GM atrophy between carriers of key mutations known to cause FTD with similar patterns (albeit to a lesser extent) seen during the presymptomatic phase. We found decreases in gray matter in presymptomatic participants that survived stringent correction for multiple comparisons: both within *C9orf72* carriers in the thalamus, cerebellar crus, frontal, and temporal lobes, as well as in the anterior insula across all presymptomatic carriers. Further studies are required not only to increasingly stratify the presymptomatic cohort but also of other imaging modalities including diffusion tensor imaging and resting-state functional MRI to understand how the GM regions identified in this study are structurally and functionally connected, allowing insight into the earliest involved areas in genetic FTD and how disease propagates from those regions.

## Disclosure statement

The authors report no conflicts of interest.
