# Supplementary material for: Patterns of gray matter atrophy in genetic frontotemporal dementia: results from the GENFI study
Source: Neurobiol Aging. 2018 Feb;62:191–6. doi: 10.1016/j.neurobiolaging.2017.10.008 (PMC5759893; doi:10.1016/j.neurobiolaging.2017.10.008)
Supplement: Supplementary Figure 1 and Supplementary Tables 1–3 [file mmc1.docx]

eFigure 1 – Top row – Significant findings of decreased GM in affected carriers (all mutations) compared to non-carriers, thresholded at p<0.05 (FWE corrected). Bottom row – Significant findings of decreased GM in presymptomatic carriers (all mutations) compared to non-carriers, thresholded at an uncorrected p value of p<0.001. The finding within the right anterior insula that did survive multiple comparisons has been circled in the bottom left hand image (axial slice at z=-8).

eTable 1 – Significant results of each of the contrasts tested in model 1. In order to limit the size of the table, we have only shown results who cluster size are greater than the value expected for a given t-statistic threshold. Findings that survive correction for multiple comparisons are shown in bold.

eTable 2 – Significant results of each of the contrasts tested in model 2 (affected participants). In order to limit the size of the table, we have only shown results who cluster size are greater than the value expected for a given t-statistic threshold. Findings that survive correction for multiple comparisons are shown in bold.

eTable 3 – Significant results of each of the contrasts tested in model 2 (presymptomatic participants). In order to limit the size of the table, we have only shown results who cluster size are greater than the value expected for a given t-statistic threshold. Findings that survive correction for multiple comparisons are shown in bold.

**eFigure 1** – **Top row – Significant findings of decreased GM in affected carriers (all mutations) compared to non-carriers, thresholded at p<0.05 (FWE corrected). Bottom row – Significant findings of decreased GM in presymptomatic carriers (all mutations) compared to non-carriers, thresholded at an uncorrected p value of p<0.001. The finding within the right anterior insula that did survive multiple comparisons has been circled in the bottom left hand image (axial slice at z=-8).**

**
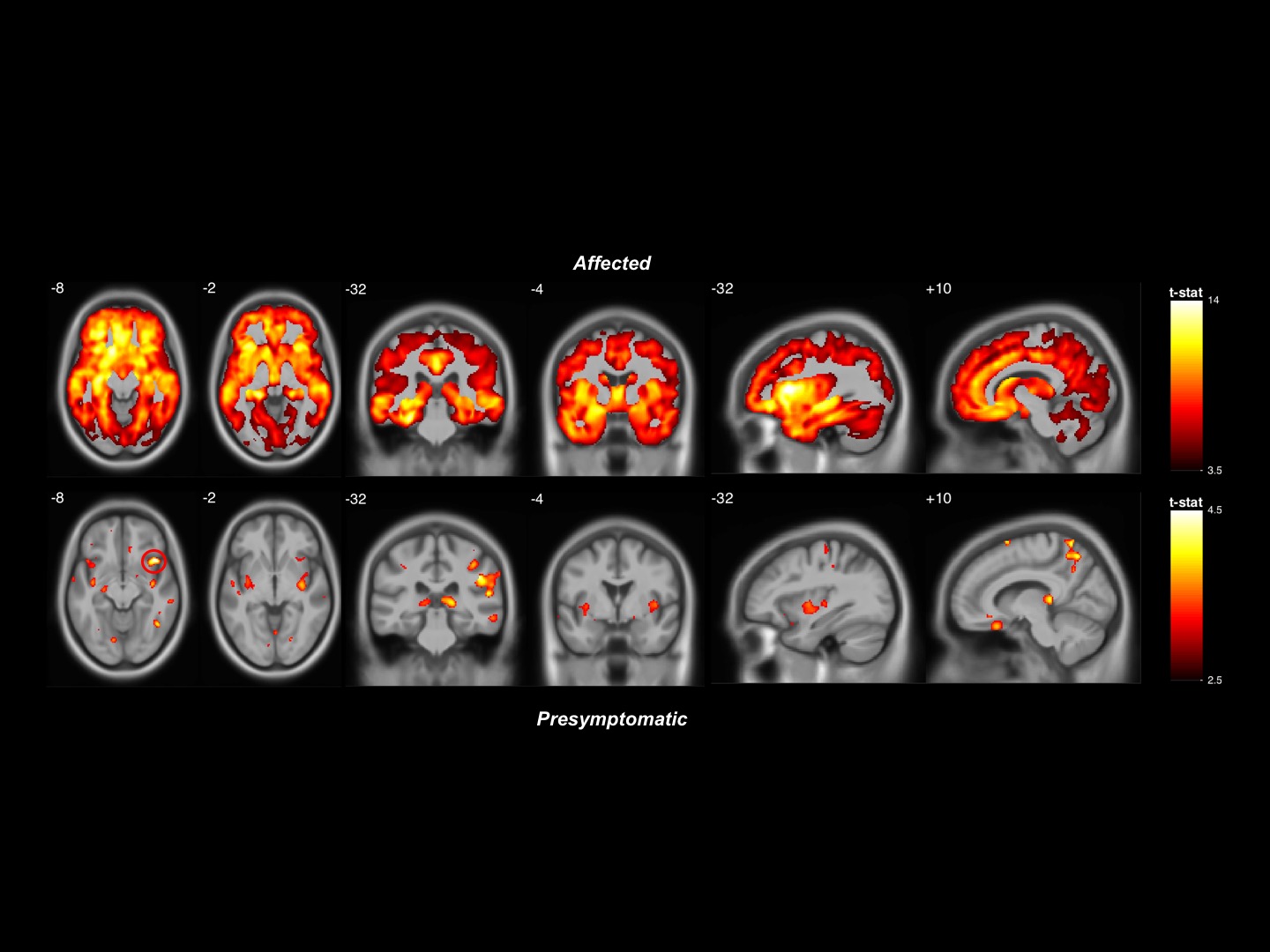
**

**eTable 1 – Findings from the contrasts tested in model 1, with those that survive correction for multiple comparisons shown in bold. For affected carriers, only results that survived stringent correction of multiple comparisons using family wise error (FWE) rate at a level of p<0.05 were included, while for presymptomatic carriers we provide all findings at an uncorrected level of p<0.001 in order to better characterize the pattern of atrophy that does not reach statistical significance. Only those findings with a cluster extent greater than or equal to the expected size for the t-statistic threshold were included.**

| **Brain Regions within cluster** | **Cluster Size** | **Peak t-statistic**  **(peak p-val, FWE corrected)** |
| --- | --- | --- |
| **Affected carriers < Non carriers** | | |
| **Insula, orbitofrontal cortex, inferior frontal lobe, thalamus, striatum, cingulate, temporal lobe, parietal lobe, cerebellar crus** | **878300** | **14.57 (p<0.001)** |
| **Cerebellum** | **448.9** | **5.75 (p<0.001)** |
| **Presymptomatic carriers < Non carriers** | | |
| **Right anterior insula** | **1077** | **4.76 (p=0.039)** |
| Right parietal operculum, pars triangularis, supramarginal gyrus | 1920 | 4.48 |
| Right precuneus | 2690 | 4.48 |
| Right angular gyrus | 1691 | 4.40 |
| Left temporal pole | 293.6 | 4.31 |
| Right fusiform gyrus | 496.1 | 4.24 |
| Right postcentral gyrus | 415.1 | 4.22 |
| Right thalamus | 1080 | 4.06 |
| Left supramarginal gyrus, planum temporale | 371.3 | 4.01 |
| Right postcentral gyrus | 192.4 | 3.92 |
| Right posterior insula | 1715 | 3.92 |
| Right postcentral gyrus | 556.9 | 3.88 |
| Left planum temporale, superior temporal gyrus | 313.9 | 3.84 |
| Right gyrus rectus, subcallosal area, medial orbital gyrus | 806.6 | 3.84 |
| Left posterior insula | 1536 | 3.80 |
| Left angular gyrus | 209.3 | 3.78 |
| Left precentral gyrus | 523.1 | 3.76 |
| Left thalamus | 465.8 | 3.73 |
| Right inferior frontal gyrus (opercular part) | 317.3 | 3.72 |
| Left anterior insula | 425.3 | 3.63 |
| Right medial temporal gyrus | 229.5 | 3.59 |

**eTable 2 – Findings from the contrast tested in model 2 involving affected participants, with those that survive correction for multiple comparisons shown in bold. For contrasts between affected participants and non carriers, only results that survived stringent correction of multiple comparisons using family wise error (FWE) rate at a level of p<0.05 were included. For comparisons between affected subgroups we provide all findings at an uncorrected level of p<0.001 in order to better characterize the pattern of atrophy that does not reach statistical significance. Only those findings with a cluster extent greater than or equal to the expected size for the t-statistic threshold were included.**

| **Brain Regions within cluster** | **Cluster Volume (mm^3^)** | **Peak t-statistic**  **(peak p-val, FWE corrected)** |
| --- | --- | --- |
| **Affected *C9orf72 <* Non carriers** | | |
| **Thalamus, insula, cingulate, frontal lobe (orbitofrontal, prefrontal), hippocampus, temporal lobe (fusiform, parahippocampal gyrus, temporal pole), parietal lobe (precuneus), occipital lobe, cerebellar crus** | **700700** | **12.46 (p<0.001)** |
| **Left cerebellum** | **1272** | **6.37 (p<0.001)** |
| **Right precentral gyrus** | **101.3** | **5.18 (p=0.007)** |
| **Left angular gyrus, supramarginal gyrus** | **57.38** | **5.11 (p=0.009)** |
| **Affected *GRN <* Non carriers** | | |
| **Caudate, putamen, frontal lobe (prefrontal), cingulate, insula, precuneus** | **3222000** | **11.86 (p<0.001)** |
| **Right angular gyrus** | **5734** | **7.11(p<0.001)** |
| **Right fusiform gyrus, inferior temporal gyrus** | **4253** | **6.91 (p<0.001)** |
| **Right cerebellum** | **4121** | **6.00 (p<0.001)** |
| **Left fusiform gyrus, inferior temporal gyrus** | **1121** | **5.88(p<0.001)** |
| **Right angular gyrus** | **148.5** | **5.66(p=0.001)** |
| **Right hippocampus** | **84.38** | **5.62(p=0.001)** |
| **Right superior parietal lobule** | **266.6** | **5.47(p=0.002)** |
| **Right middle temporal gyrus** | **111.4** | **5.46(p=0.002)** |
| **Left inferior temporal gyrus** | **205.9** | **5.27(p=0.005)** |
| **Left middle temporal gyrus** | **101.3** | **5.21(p=0.006)** |
| **Left fusiform gyrus, lingual gyrus** | **216.0** | **5.19(p=0.006)** |
| **Right hippocampus** | **108.0** | **5.14(p=0.008)** |
| **Right temporal pole** | **138.4** | **5.11(p=0.009)** |
| **Right operculum** | **74.25** | **5.10(p=0.01)** |
| **Left cerebellum** | **1242** | **5.07(p=0.011)** |
| **Right thalamus, hippocampus** | **131.6** | **5.05(p=0.012)** |
| **Left parietal operculum, planum temporale** | **97.88** | **5.01(p=0.014)** |
| **Left postcentral gyrus** | **70.88** | **5.00(p=0.015)** |
| **Affected *MAPT* < Non carriers** | | |
| **Temporal lobe, insula, hippocampus, amygdala, orbitofrontal cortex, anterior cingulate, nucleus accumbens** | **283900** | **14.16(p<0.001)** |
| **Middle/posterior cingulate, precuneus** | **11540** | **7.04(p<0.001)** |
| **Left superior frontal gyrus** | **1569** | **6.10(p<0.001)** |
| **Right angular gyrus** | **924.8** | **5.87(p<0.001)** |
| **Right frontal pole** | **212.6** | **5.70(p=0.001)** |
| **Lingual gyrus** | **354.4** | **5.68(p=0.001)** |
| **Left postcentral gyrus, supramarginal gyrus** | **796.5** | **5.63(p=0.001)** |
| **Left precuneus, superior parietal lobule** | **175.5** | **5.61(p=0.001)** |
| **Right superior frontal gyrus** | **361.1** | **5.59(p=0.001)** |
| **Left supplementary motor cortex** | **148.5** | **5.44(p=0.002)** |
| **Right occipital lobe (calcarine cortex, cuneus, superior occipital gyrus)** | **108.0** | **5.38(p=0.003)** |
| **Left middle frontal gyrus** | **108.0** | **5.35(p=0.003)** |
| **Left middle frontal gyrus** | **111.4** | **5.27(p=0.005)** |
| **Left middle frontal gyrus** | **381.4** | **5.27(p=0.005)** |
| **Right lingual gyrus** | **60.75** | **5.26(p=0.005)** |
| **Right lingual gyrus** | **74.25** | **5.20(p=0.006)** |
| **Right middle frontal gyrus** | **739.1** | **5.19(p=0.006)** |
| **Right superior frontal gyrus, middle frontal gyrus** | **178.9** | **5.16(p=0.007)** |
| **Right superior occipital gyrus** | **151.9** | **5.12(p=0.009)** |
| **Left angular gyrus, supramarginal gyrus, superior parietal lobule,** | **131.6** | **5.10(p=0.01)** |
| **Right supramarginal gyrus** | **64.12** | **5.10(p=0.01)** |
| **Left precuneus** | **57.38** | **4.95(p=0.018)** |
| **Affected: *C9orf72* < *GRN*** | | |
| **Bilateral fusiform gyrus, lingual gyrus, calcarine cortex** | **8836** | **4.83(p=0.029)** |
| **Left hippocampus, parahippocampal gyrus, entorhinal cortex, amygdala** | **5221** | **4.71(p=0.047)** |
| Right thalamus | 843.8 | 4.07 |
| Left temporal pole | 519.8 | 3.91 |
| Left inferior temporal gyrus | 367.9 | 3.76 |
| Occipital lobe (calcarine cortex, cuneus, lingual gyrus) | 256.5 | 3.69 |
| Left superior temporal gyrus | 391.5 | 3.66 |
| Right hippocampus | 202.5 | 3.43 |
| Right hippocampus | 256.5 | 3.29 |
| **Affected: *C9orf72* < *MAPT*** | | |
| Left lingual gyrus, cerebellum | 276.8 | 3.88 |
| Right thalamus | 567.0 | 3.72 |
| Right fusiform gyrus, cerebellum | 324.0 | 3.53 |
| Left fusiform gyrus, cerebellum | 192.4 | 3.46 |
| **Affected: *GRN < C9orf72*** | | |
| **Left dorsal striatum** | **6953** | **6.46(p<0.001)** |
| **Left middle frontal gyrus, superior frontal gyrus, precentral gyrus** | **3351** | **5.02(p=0.014)** |
| **Right dorsal striatum** | **3652** | **4.92(p=0.020)** |
| **Left precentral gyrus** | **1644** | **4.90(p=0.022)** |
| Left superior frontal gyrus | 307.1 | 4.05 |
| Left superior frontal gyrus, supplementary motor cortex | 607.5 | 3.78 |
| Right precentral gyrus | 266.6 | 3.42 |
| **Affected: *GRN* < *MAPT*** | | |
| **Left dorsal striatum** | **4178** | **5.95(p<0.001)** |
| **Left middle frontal gyrus, precentral gyrus** | **3578** | **4.89(p=0.023)** |
| Right dorsal striatum | 1691 | 4.47 |
| Right precentral gyrus | 2140 | 4.08 |
| Supplementary motor cortex, medial precentral gyrus | 735.8 | 3.93 |
| Right superior frontal gyrus, middle frontal gyrus | 526.5 | 3.81 |
| Left thalamus | 543.4 | 3.75 |
| **Affected: *MAPT* < *C9orf72*** | | |
| **Right posterior insula, amygdala, hippocampus, inferior/medial temporal lobe** | **31750** | **6.83(p<0.001)** |
| **Left posterior insula, amygdala, hippocampus, inferior/medial temporal lobe** | **23290** | **6.53(p<0.001)** |
| Right nucleus accumbens | 644.6 | 4.45 |
| Right hippocampus (tail) | 388.1 | 4.37 |
| Left nucleus accumbens | 783.0 | 4.15 |
| Left hippocampus (tail) | 219.4 | 3.65 |
| Right middle temporal gyrus | 243.0 | 3.56 |
| **Affected: *MAPT* < *GRN*** | | |
| **Left amygdala, hippocampus, inferior/medial temporal lobe, posterior insula** | 43330 | **8.75(p<0.001)** |
| **Right amygdala, hippocampus, inferior/medial temporal lobe, posterior insula** | 44830 | **8.37(p<0.001)** |
| Right nucleus accumbens | 614.3 | 4.25 |

**eTable 3 – Findings from the contrast tested in model 2 involving presymptomatic participants, with those that survive correction for multiple comparisons shown in bold. In all contrasts shown in this table, we provide findings at an uncorrected level of p<0.001 in order to better characterize the pattern of atrophy that does not reach statistical significance using FWE. Only those findings with a cluster extent greater than or equal to the expected size for the t-statistic threshold were included.**

| **Brain Regions within cluster** | **Cluster Size** | **Peak t-statistic**  **(peak p-val, FWE corrected)** |
| --- | --- | --- |
| **Presymptomatic *C9orf72* < Non carriers** | | |
| **Bilateral thalamus** | **12090** | **6.08 (p<0.001)** |
| **Right cerebellum** | **12990** | **4.98 (p=0.016)** |
| **Right pars triangularis, inferior frontal gyrus** | **1492** | **4.79 (p=0.035)** |
| **Left superior temporal gyrus, transverse temporal gyrus, planum polare, planum temporale** | **1688** | **4.71 (p=0.048)** |
| Left precentral gyrus, postcentral gyrus, supramarginal gyrus, superior parietal lobule | 4350 | 4.51 |
| Left cerebellum | 2112 | 4.47 |
| Right postcentral gyrus, supramarginal gyrus, superior parietal lobule, precentral gyrus | 1823 | 4.32 |
| Left precentral gyrus, opercular part inferior frontal gyrus, frontal operculum | 1559 | 4.31 |
| Right planum temporale, parietal operculum, supramarginal gyrus, superior temporal gyrus | 1637 | 4.27 |
| Bilateral precuneus, right superior parietal lobule | 1927 | 4.25 |
| Right lingual gyrus, cerebellum, vermis lobules I-V, occipital fusiform gyrus | 3345 | 4.18 |
| Left cerebellum, fusiform gyrus | 7226 | 4.14 |
| Left supramarginal gyrus, planum temporale, parietal operculum | 1667 | 4.09 |
| Right middle occipital gyrus, superior occipital gyrus, superior parietal lobule | 513.0 | 4.09 |
| Left cerebellum | 465.8 | 4.05 |
| Left pars triangularis, lateral orbitofrontal cortex | 276.8 | 3.98 |
| Right fusiform, inferior temporal gyrus | 354.4 | 3.94 |
| Right insula, posterior orbital gyrus, temporal pole | 594.0 | 3.93 |
| Bilateral gyrus rectus, medial frontal cortex | 1188 | 3.93 |
| Left middle temporal gyrus, superior temporal gyrus | 327.4 | 3.92 |
| Right cerebellum | 273.4 | 3.85 |
| Right cerebellum | 1033 | 3.79 |
| Left anterior cingulate | 317.3 | 3.71 |
| Right precuneus, posterior cingulate | 496.1 | 3.67 |
| Right hippocampus | 195.8 | 3.64 |
| Left anterior insula, frontal operculum, inferior frontal gyrus, posterior orbital gyrus | 1006 | 3.64 |
| Left lingual gyrus, precuneus, calcarine cortex | 239.6 | 3.64 |
| Bilateral posterior cingulate | 340.9 | 3.56 |
| Left middle frontal gyrus, superior frontal gyrus | 259.9 | 3.51 |
| Right fusiform gyrus, inferior temporal gyrus | 202.5 | 3.50 |
| **Presymptomatic *GRN* < Non carriers** | | |
| Right parietal operculum, supramarginal gyrus | 448.9 | 4.44 |
| Right inferior frontal gyrus | 226.1 | 3.50 |
| Right posterior insula, putamen, superior temporal | 273.4 | 3.48 |
| **Presymptomatic *MAPT* < Non carriers** | | |
| Right inferior temporal gyrus, fusiform gyrus | 850.5 | 4.24 |
| Right amygdala, hippocampus, entorhinal cortex | 1586 | 3.79 |
| Left amygdala, hippocampus, entorhinal cortex, orbitofrontal lobe | 509.6 | 3.71 |
| Right angular gyrus, superior parietal lobule | 580.5 | 3.70 |
| Right inferior temporal gyrus, middle temporal gyrus | 280.1 | 3.67 |
| Right middle temporal gyrus | 216.0 | 3.66 |
| **Presymptomatic:  *C9orf72 < GRN*** | | |
| Bilateral thalamus, left hippocampus | 4566 | 5.09 |
| Right thalamus, hippocampus | 1370 | 4.58 |
| Left inferior frontal gyrus, precentral gyrus, operculum | 1755 | 4.22 |
| Left precentral gyrus | 351.0 | 3.86 |
| Right cerebellum | 644.6 | 3.75 |
| Left cerebellum | 273.4 | 3.71 |
| Right lingual gyrus | 266.6 | 3.50 |
| Left precuneus, calcarine cortex | 209.3 | 3.50 |
| **Presymptomatic:  *C9orf72* < *MAPT*** | | |
| Bilateral thalamus | 317.3 | 4.03 |
| Left superior frontal gyrus, supplementary motor cortex | 789.8 | 4.02 |
| Right cerebellum | 1971.0 | 3.99 |
| Right cerebellum | 770.0 | 3.55 |
| Right cerebellum | 205.9 | 3.34 |
| **Presymptomatic: *GRN* < *C9orf72*** | | |
| NO FINDINGS | | |
| **Presymptomatic: *GRN* < *MAPT*** | | |
| NO FINDINGS | | |
| **Presymptomatic: *MAPT* < *C9orf72*** | | |
| NO FINDINGS | | |
| **Presymptomatic: *MAPT* < *GRN*** | | |
| NO FINDINGS | | |
